# Local adaptation and evolutionary potential along a temperature gradient in the fungal pathogen *Rhynchosporium commune*

**DOI:** 10.1111/eva.12039

**Published:** 2013-01-03

**Authors:** Tryggvi S Stefansson, Bruce A McDonald, Yvonne Willi

**Affiliations:** 1 Institute of Integrative Biology, Plant Pathology, ETH ZürichZürich, Switzerland; 2 Institute of Biology, Evolutionary Botany, University of NeuchâtelNeuchâtel, Switzerland

**Keywords:** growth rate, G × E, heritability, *Q*_ST_/*G*_ST_, thermal adaptation, thermal stress

## Abstract

To predict the response of plant pathogens to climate warming, data are needed on current thermal adaptation, the pathogen's evolutionary potential, and the link between them. We conducted a common garden experiment using isolates of the fungal pathogen *Rhynchosporium commune* from nine barley populations representing climatically diverse locations. Clonal replicates of 126 genetically distinct isolates were assessed for their growth rate at 12°C, 18°C, and 22°C. Populations originating from climates with higher monthly temperature variation had higher growth rate at all three temperatures compared with populations from climates with less temperature fluctuation. Population differentiation in growth rate (*Q*_ST_) was significantly higher at 22°C than population differentiation for neutral microsatellite loci (*G*_ST_), consistent with local adaptation for growth at higher temperatures. At 18°C, we found evidence for stabilizing selection for growth rate as *Q*_ST_ was significantly lower than *G*_ST_. Heritability of growth rate under the three temperatures was substantial in all populations (0.58–0.76). Genetic variation was lower in populations with higher growth rate at the three temperatures and evolvability increased under heat stress in seven of nine populations. Our findings imply that the distribution of this pathogen is unlikely to be genetically limited under climate warming, due to its high genetic variation and plasticity for thermal tolerance.

## Introduction

There is an ample evidence that climate warming affects the distribution of species (Parmesan 2006). The likely reason is that distributions are often strongly affected by the breadth of tolerance for temperature (e.g., for animals: Andrewartha and Birch 1954; for plants: Woodward and Williams 1987). Understanding performance under various temperatures is therefore important for predicting species distribution under environmental change (Austin 2007). However, thermal performance may not be fixed within species but may vary among populations due to adaptation to prevailing local conditions (Huey and Kingsolver 1989; Angilletta et al. 2002). Divergent thermal performance in relation to climate heterogeneity within current species distributions may provide insight into past temperature adaptation. And assessing genetic diversity parameters can reveal whether local populations will be able to track future changes in temperature, even in the absence of gene flow. According to theory, the potential to adapt to changes in temperature depends on the amount of genetic variation for tolerance toward temperature extremes (Lynch and Lande 1993) and on genetic variation for phenotypic plasticity (Chevin et al. 2010; Chevin and Lande 2011). The parallel study of both local adaptation and genetic variation within populations should reveal the potential of populations to adapt to changing temperatures.

Temperature adaptation over heterogeneous space can take several forms. Populations may shift their thermal optimum for performance to reflect the local temperature regime (Levinton 1983; Levinton and Monahan 1983). Besides, populations can exhibit co- or counter-gradient variation in environmental and genetic effects along a temperature cline (Conover and Schultz 1995). Counter-gradient variation can be identified when genotypes that encode fast growth are found in environments that would otherwise cause slow growth, for example, because of extremely low or high temperature or low food availability (Conover et al. 2009). An example of co-gradient variation is when growth is faster at warmer compared with cooler sites because of both environmental and local genetic effects (Conover et al. 2009). Examples of counter-gradient variation in aspects of performance have now been found in at least 60 species (Levins 1968, 1969; Conover et al. 2009). Despite such variation being difficult to detect in nature because it can be masked by environmental effects (Merilä et al. 2001; Wilson et al. 2007; Conover et al. 2009), the model can provide useful perspectives on certain types of thermal adaptation.

To predict future responses to selection, knowledge about the relationship between past adaptation and standing genetic variation is needed. Past selection on thermal performance can be inferred by comparing population genetic divergence in growth rate under various temperatures (*Q*_ST_) and population divergence in neutral genetic markers (*G*_ST_). Significantly higher *Q*_ST_ values than *G*_ST_ values can be interpreted as diversifying selection for local adaptation, whereas stabilizing selection is expected to decrease population differentiation in growth rate leading to lower *Q*_ST_ than *G*_ST_ (Spitze 1993). Once the prevailing type of past selection is revealed, its link to within-population genetic variation can be drawn. According to theory, directional selection lowers within-population genetic variation (e.g., Keightley and Hill 1987; reviewed in Willi et al. 2006). The traditional measure of within-population genetic variation has been heritability, defined as the fraction of total phenotypic variation attributable to additive genetic variation. Houle (1992) suggested the alternative method of scaling the additive genetic variation to the trait mean, particularly for life-history traits. A recent meta-analysis on the relationship between the two measures showed the correlation to be essentially zero across studies (Hansen et al. 2011). The mean-scaled method is especially suitable for measuring evolvability (defined as the predicted ability of a population to respond to selection) in comparisons of additive genetic variance across environments (Hansen et al. 2011). Scaling to the mean – all else being equal – should produce evolvability estimates that decrease with an evolved increase in thermal performance.

Both genetically determined phenotypic plasticity and environmental canalization (reflecting genotype-by-environment or G × E interactions) are also important for adaptation to new and changing environments and reflect another form of adaptability (Bradshaw 1965; Scheiner 1993; Ghalambor et al. 2007). Phenotypic plasticity occurs when the phenotype produced by a single genotype is sensitive to variation in the environment, whereas environmental canalization reflects insensitivity of the phenotype to environmental variation (Schlichting 2008). Canalization is expected to be favored when the optimum phenotype is the same across environments, but plasticity should be favored if the optimum phenotype differs across environments (Bradshaw 1965; Flatt 2005). The presence of significant G × E interactions has been looked at from several perspectives. First, for a fitness-related trait assessed under various environmental conditions, a significant G × E interaction may indicate that high performance cannot be maintained across a wide temperature range, which could be interpreted as the signature of a specialist or an indication of a trade-off between performance in different environments (Yamahira and Conover 2002). Second, significant G × E suggests that there is genetic variation for phenotypic plasticity or environmental canalization (Bradshaw 1965; Via and Lande 1985; Whitman and Agrawal 2009). Third, significant G × E between stressful and benign conditions has been interpreted as environment dependence of genetic variation with either positive or negative effects on genetic diversity estimates for each environment (Hoffmann and Merilä 1999).

We assessed both the extent of local adaptation and genetic variation for thermal performance in field-collected populations of the fungus *Rhynchosporium commune*. This species is a haploid ascomycete and a major pathogen of barley worldwide (reviewed in Avrova and Knogge 2012). We conducted a common garden experiment, measuring growth rate at three temperatures (12°C, 18°C, and 22°C) for 126 genetically distinct isolates from nine populations collected along a climatic gradient ranging from the tropics to the subarctic. The 10 degree temperature range was chosen to reflect ecologically relevant temperatures that the pathogen is exposed to during the growing season of the host (i.e., during the pathogenic phase of its life cycle) in all geographical regions, with 12°C and 18°C representing relatively benign temperatures experienced during rapid host development and 22°C representing a more stressful temperature experienced near the end of the host life cycle. *R. commune* has four known infection routes. It can overwinter or oversummer in the absence of a living host as a saprophyte on barley residue to infect the next generation of barley seedlings. It also can survive between barley plantings as a pathogen on volunteer barley. The pathogen sporulates on the leaf surface, with spores spreading to nearby plants via rain-splash and it can also survive on barley seeds (Shipton et al. 1974). As *R. commune* genotypes have been shown to persist in the field as a saprophyte for at least 1 year (Abang et al. 2006), we used climatic variables such as mean annual temperature and variation in mean annual temperature to describe local thermal conditions. We addressed the following primary questions and considered the experimental outcomes with regard to the distribution and adaptability of *R. commune* in relation to climate change.

1. Is there a correlation between climate gradients and growth rate, indicating climate adaptation?
2. What are the relative roles of natural selection and neutral evolution in shaping thermal performance?
3. What is the level of genetic variation for thermal performance and how does it relate with past selection and trait means?

## Material and methods

### *Rhynchosporium commune* populations

Isolates of *Rhynchosporium commune* were collected from naturally infected barley fields in nine countries on four continents (Table 1). Climate data were acquired from The International Water Management Institute (http://wcatlas.iwmi.org), and monthly temperature means were used to calculate annual mean, variance, minimum, and maximum (Table 1). Seven of the populations were used in previous studies, including Finland and Norway (Salamati et al. 2000), California, USA (McDermott et al. 1989), Australia (McDonald et al. 1999), Switzerland and Ethiopia (Linde et al. 2003), and New Zealand (Linde et al. 2009). Two of these populations had not been described previously. The Icelandic population was collected in August 2008 from a barley field in the south of Iceland, near the experimental research station Korpa. The Spanish population was collected in May 2005 from Aranjuez in the Tagus river valley in central Spain.

**Table 1:** The nine populations of *Rhynchosporium commune* included in the experiment. Country of origin, field location, latitude, longitude, mean annual temperature in °C (MAT), variance in mean annual temperature (varMAT), as well as minimum and maximum monthly mean temperatures (Min/Max)

Country	Location	Latitude	Longitude	MAT	varMAT	Min	Max
Ethiopia (ET)	Adineba	12.28°N	39.20°E	16.3	2.3	14.0	18.6
USA (US)	Davis, CA	38.33°N	121.44°W	15.7	36.4	7.2	23.6
Australia (AU)	Rannock	34.36°S	147.15°E	15.5	36.3	7.4	23.7
Spain (SP)	Aranjunez	40.2°N	3.36°W	14.1	50.1	5.7	24.8
New Zealand (NZ)	Rakaia	43.45°S	172.1°E	11.5	16.9	5.7	17.0
Switzerland (CH)	Cugy	46.38°N	6.38°E	8.7	44.5	–0.5	17.7
Norway (NO)	Buskerud	59.33°N	11.19°E	5.5	58.4	–4.2	15.9
Finland (FI)	Jokioinen	60.48°N	23.29°E	3.8	76.3	–7.8	15.7
Iceland (IS)	Reykjavik	64.9°N	21.45°W	3.6	19.3	–1.5	10.1

### Colony growth rate at different temperatures

*In vitro* growth of isolates was assessed at 12°C, 18°C, and 22°C. Fourteen genetically distinct isolates were chosen at random from each of the nine populations and revived from -80°C storage by plating on Lima Bean Agar (LBA, 60 g/L Lima Beans, 12 g/L Agar, 50 mg/L kanamycin). Following 10–14 days of growth at 18°C in darkness, isolates were transferred to fresh LBA plates and grown for 2 weeks. Spores were harvested from plates by adding 1.5 ml of sterile water and scraping with a sterile microscope slide. Spores were counted using a hemocytometer and diluted to a concentration of 1000 spores/mL. One hundred microliters of spore solution was placed on nine standardized 60 mm Potato Dextrose Agar plates (PDA, 24 g/L Potato Dextrose, 15 g/L Agar, 50 mg/L kanamycin) per isolate and spores were spread with a sterile glass rod. To minimize systematic differences in the treatment of isolates, the growth medium was prepared as a single batch that was poured into Petri plates on the same day, the Petri plates were randomized before inoculation, and isolates from different populations were randomized before inoculation onto the plates. Following inoculation, plates were left to dry for 15–20 min and then were incubated in darkness at 12°C, 18°C, and 22°C, with three replicates per isolate and temperature. Fungal colonies on plates were photographed with a digital camera after 12, 15, 18, 21, 24, 34, and 44 days of growth under standardized conditions, and the colony diameter (mm) and size (mm^2^) were measured with the image analysis software APS Assess 2.0 (Lamari 2002). The growth trajectory was first assessed by plotting log-transformed mean colony size against log-transformed time (number of days). Data showed that across isolates and populations, the power function was linear between day 12 and day 24. Therefore, growth rate was calculated for each individual replicate as the steepness of the linear power function slope between days 12 and 24 (SAS Institute 2008, PROC REG).

### Microsatellite genotyping

All isolates were genotyped using eight microsatellite loci: Rh1, Rh2, Rh4, Rh5, Rh6, Rh8*, Rh11, and Rh14 using fluorescently labeled primers and conditions as previously described (Linde et al. 2005). Alleles were assigned using the software GeneMapper 4.1 (Applied Biosystems). Linde et al. (2009) tested the loci for selective neutrality using the Ewens–Watterson test (Smouse and Neel 1977; Yang and Yeh 1993) and did not find any deviations of haplotype distributions from neutral expectations. Population differentiation for the microsatellite loci (*G*_ST_) was assessed both pairwise and overall using the software FSTAT 2.9.3 (Goudet 1995). Standard deviations for overall *G*_ST_ values were calculated by jackknifing over loci.

### Data analysis

To determine whether growth rate differed among isolates, populations, and temperatures, a general linear model analysis was performed using SAS software (SAS Institute 2008; PROC GLM) to test the following effects: isolate/genotype (G) nested within population, population (P), temperature (T), and interactions with temperature. The full model was Y = M + G(P) + P + T + T × G(P) + T × P + E. Y referred to the growth rate of replicate R for isolate I in population P at temperature T, and M was the overall mean. Temperature-independent environmental effects were measured as the variance between replicates (E). This is possible because in a common garden experiment with asexually reproducing species, variance between replicates can be attributed mostly to temperature-independent environmental effects because different replicates have the same genotype, that is, they are clones (Zhan et al. 2005).

To evaluate the importance of local adaptation in thermal tolerance, a comparison was made of population divergence at microsatellite loci (*G*_ST_) with divergence for growth rate at different temperatures (*Q*_ST_). Population differentiation for growth rate was calculated as the ratio of additive genetic variation among populations to the sum of additive genetic variation within and between populations (Spitze 1993; Zhan et al. 2005). This analysis was performed at each temperature separately using the model Y = M + G(P) + P + E. *Q*_ST_ values were calculated for all pairwise combinations of populations as well as across all populations (SAS Institute 2008; PROC MIXED, method = reml). Standard errors for overall *Q*_ST_ values were obtained by jackknifing over populations (Miller 1968; Knapp et al. 1989). A paired *t*-test was used to determine if differences between pairwise *Q*_ST_ values for each temperature and pairwise *G*_ST_ values were significant.

Genetic variation for growth rate was assessed using three different parameters. First, it was measured as the additive genetic variation (*V*_G_), the variance explained by isolates within populations. For a haploid species in a common garden environment, if epistasis is negligible, then phenotypic variance within a population consists only of additive genetic variance and temperature-independent environmental variance (residual variance) because dominance does not exist. Second, heritability (*h*^2^) was calculated as the ratio of additive genetic variance to total phenotypic variance within populations. Third, trait mean-standardized measures of additive genetic variance (*I*_G_) were used for comparisons across temperatures (Houle 1992). Variance components were calculated within each environment and population using the model Y = M + G + E (SAS Institute 2008, PROC MIXED, method = reml). The standard errors of variance components were obtained by jackknifing over isolates within each population and environment. Levels of mean-standardized additive genetic variation under heat stress (22°C) were compared with those under 18°C by taking the log-transformed ratio of mean-standardized additive genetic variation at 22°C compared with 18°C. The decrease in growth rate between 18°C and 22°C was quantified by calculating the mean slope of the reaction norm between the two temperatures for each population based on isolate means.

Genotype-by-temperature interactions (equivalent to genotype-by-environment interaction, G × E) were calculated for each population using the model Y = M + G + T + G × T + E (SAS Institute 2008, PROC MIXED, method = reml). Analysis was done for two temperature combinations, 12–18°C and 18–22°C, and the standard errors of variance components were obtained by jackknifing over isolates within each population and temperature combination. Plasticity was estimated by calculating the slope of reaction norms between temperatures for each isolate. Plasticity levels for each population were then calculated as the mean slope, and standard deviations were estimated based on variation between isolate slopes.

Correlation analyses were used to test for the following associations: (i) population-mean growth rate between the three experimental temperatures, (ii) population-mean growth rate at each temperature and climatic conditions at collection sites, (iii) estimates of genetic variation and population-mean growth rate, and (iv) plasticity estimates and population-mean growth rate. Significance was based on two-tailed *P*-values. A two-tailed Students *t*-test was used to compare differences in mean growth rate between populations and temperatures and overall growth rate between temperatures (Sokal and Rohlf 1995).

## Results

**Figure 1:**
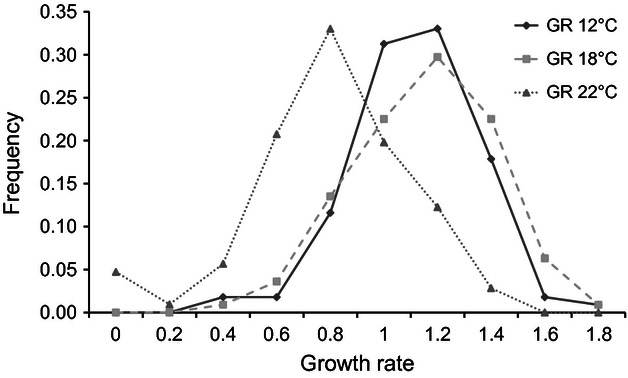
Frequency distribution of growth rates under the three experimental temperatures for all 126 isolates of *Rhynchosporium commune* from all nine populations.

Populations and isolates within populations differed significantly in growth rate and there was a significant interaction between them and temperature (Table 2). Temperature also significantly impacted growth rate. Histograms for growth rate showed that they were not very different between 12°C and 18°C but were significantly lower at 22°C compared with 12°C (df = 204, *t* = 9.16, *P* ≤ 0.001) and 18°C (df = 211, *t* = 9.64, *P* ≤ 0.001) (Fig. 1). For all nine populations, mean growth rate was lowest at 22°C (Table 3) and only 4% of the isolates included in the study had the highest growth rate at 22°C. The highest mean growth rate was at 12°C for the populations from California and Finland: 86% of the California isolates and 71% of the Finnish isolates had the highest growth rate at 12°C. The highest mean growth rate for the other populations was at 18°C, although mean growth rate was only significantly higher at 18°C compared with 12°C in the population from Spain (df = 19, *t* = 3.06, *P* ≤ 0.006) where 80% of the isolates had highest growth rate at 18°C. Correlation of isolate mean growth rate between temperatures within populations revealed that there was a significant positive correlation in three populations between growth rate at 12°C and 18°C (*P* ≤ 0.006); all other populations showed nonsignificant correlations. Growth rate at 22°C was not significantly correlated with growth rate at 12°C or 18°C in any of the populations.

**Table 2:** General linear model testing the effects of isolate (nested within population), population, temperature, and their interactions on growth rate of *Rhynchosporium commune*

Source	df	MS	*F*	*P*
Isolate (pop.)	109, 548	2.5803	13.39	< 0.0001
Population	8, 109	12.8836	4.99	< 0.0001
Temperature	2, 16	87.7930	30.20	< 0.0001
Pop. × temp.	16, 193	2.9069	3.42	< 0.0001
Pop. × temp. × isol.	193, 548	0.8496	4.41	< 0.0001
Error	548	0.1927		

The effect of population was tested over isolates nested within population. Temperature was tested over its interaction with population, and that interaction was tested over the interaction including isolate. Other effects were tested over the pooled error.

**Table 3:** Least square means (LSM), additive genetic variance (*V*_G_), environmental variance (*V*_E_), and heritability (*h*^2^) for growth under three experimental temperatures for nine populations of *Rhynchosporium commune*. Standard errors (SE) for all values and countries are reported on the second line; SE for variance components came from jackknifing over isolates. The last lines summarize means, standard deviations (SD), and coefficients of variation (CV)

Country	12°C	18°C	22°C
		
	LSM	*V*_G_	*V*_E_	*h*^2^	LSM	*V*_G_	*V*_E_	*h*^2^	LSM	*V*_G_	*V*_E_	*h*^2^
CH	1.0515	0.0741	0.0168	0.8123	1.0654	0.1016	0.0104	0.9065	0.9002	0.0416	0.0166	0.7123
	0.0982	0.0029	0.0004	0.0087	0.1087	0.0026	0.0002	0.0030	0.0733	0.0016	0.0008	0.0157
US	1.0383	0.0142	0.0173	0.4481	0.9504	0.0349	0.0113	0.7513	0.6075	0.0767	0.0091	0.8913
	0.0491	0.0006	0.0003	0.0148	0.0656	0.0014	0.0003	0.0126	0.0959	0.0032	0.0002	0.0071
FI	1.1934	0.0139	0.0214	0.3904	1.0909	0.0137	0.0328	0.2936	0.9563	0.0258	0.0235	0.5209
	0.0502	0.0008	0.0006	0.0198	0.0521	0.0008	0.0008	0.0149	0.0708	0.0011	0.0005	0.0119
ET	0.9877	0.0464	0.0152	0.7456	1.0348	0.0751	0.0266	0.7373	0.3315	0.0974	0.0025	0.9748
	0.0758	0.0025	0.0004	0.0178	0.0963	0.0022	0.0010	0.0120	0.1045	0.0030	0.0002	0.0016
NO	1.1333	0.0216	0.0094	0.6958	1.1571	0.0259	0.0361	0.4167	0.8176	0.0256	0.0178	0.5885
	0.0523	0.0005	0.0001	0.0056	0.0677	0.0009	0.0007	0.0115	0.0592	0.0007	0.0004	0.0099
NZ	0.8819	0.0326	0.0170	0.6570	0.9077	0.0901	0.0154	0.8526	0.6221	0.0199	0.0098	0.6670
	0.0687	0.0008	0.0005	0.0099	0.1029	0.0029	0.0005	0.0072	0.0504	0.0009	0.0003	0.0162
SP	1.0124	0.0000	0.0471	0.0012	1.2459	0.0134	0.0394	0.2510	0.6228	0.0805	0.0170	0.8194
	0.0646	0.0000	0.0019	0.0012	0.0540	0.0010	0.0010	0.0160	0.1031	0.0063	0.0007	0.0173
IS	0.8863	0.0340	0.0199	0.6229	0.9276	0.0812	0.0119	0.8691	0.6071	0.0448	0.0053	0.8916
	0.0668	0.0019	0.0005	0.0224	0.0972	0.0037	0.0003	0.0085	0.0742	0.0015	0.0002	0.0056
AU	0.8287	0.0741	0.0141	0.8355	1.0536	0.0035	0.0265	0.1152	0.6071	0.0214	0.0051	0.8160
	0.0952	0.0094	0.0016	0.0063	0.0355	0.0008	0.0013	0.0284	0.0475	0.0028	0.0014	0.0213
Mean	1.0015	0.0345	0.0198	0.5787	1.0481	0.0488	0.0234	0.5770	0.6747	0.0482	0.0119	0.7647
SD	0.1203	0.0262	0.0108	0.2637	0.1102	0.0379	0.0114	0.3065	0.1896	0.0293	0.0071	0.1519
CV	0.1201	0.7571	0.5450	0.4556	0.1051	0.7762	0.4873	0.5312	0.2810	0.6079	0.6017	0.1987

**Figure 2:**
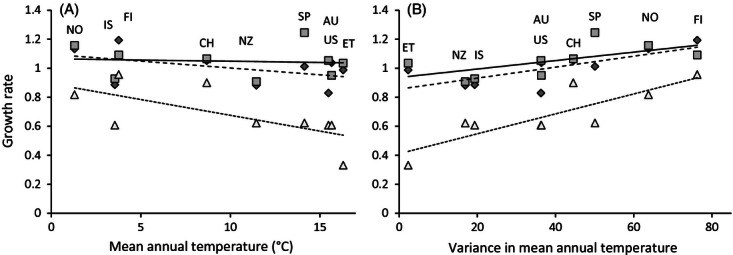
Mean growth rate in relation to the environmental variables of mean annual temperature (MAT, panel A) and variance in mean annual temperature (varMAT, panel B) for nine populations of *Rhynchosporium commune*. Symbols represent population means at the three experimental temperatures: 12°C: diamonds, 18°C: squares, and 22°C: triangles. The regression lines for each temperature are indicated: 12°C: dashed, 18°C: solid, and 22°C: dotted. Populations are abbreviated by their country's name. Correlations between population-mean growth rate and MAT were significantly negative at 22°C, and those between population-mean growth rate and varMAT were significantly positive at 12°C and 22°C and close to significance at 18°C (for statistics see Results).

Because populations differed significantly in their response to temperature, the relationship between growth rate and environmental field data was investigated on population means. As the two climatic variables, mean annual temperature and variance in monthly mean temperature, were not significantly correlated (*n* = 9, *r* = −0.46, *P* = 0.21), we explored both of them in the correlations analyses. Population-mean growth rate and mean annual temperature were not significantly correlated at 12°C (*n* = 9, *r* = −0.41, *P* = 0.27) and 18°C (*n* = 9, *r* = 0.0003, *P* = 0.99), but they were negatively associated at 22°C (*n* = 9, *r* = −0.69, *P* ≤ 0.04) (Fig. 2A). Correlations between population-mean growth rate and variance in monthly mean temperature at the field sites were significantly positive at 12°C and 22°C and close to significance at 18°C (*n* = 9, 12°C: *r* = 0.73, *P* ≤ 0.03; 18°C: *r* = 0.61, *P* = 0.08; 22°C: *r* = 0.85, *P* ≤ 0.004) (Fig. 2B). On average, isolates from populations with more variable climates had higher mean growth rate at all three temperatures than isolates from populations with more stable climates.

**Figure 3:**
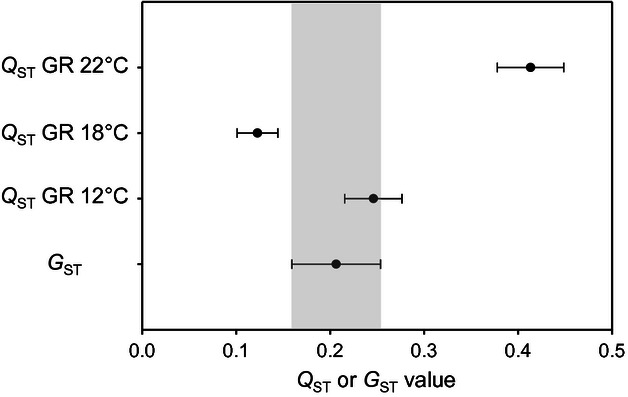
Comparison of population divergence based on neutral microsatellite loci (*G*_ST_) and population divergence in growth rate (*Q*_ST_) at the three experimental temperatures. When *Q*_ST_ exceeds *G*_ST_, this can be taken as evidence for the past action of diversifying selection. Growth rate (GR) at the highest temperature, 22°C, showed evidence for this type of selection. Error bars represent 95% confidence intervals.

A *Q*_ST_-*G*_ST_ comparison was used to reveal evidence for divergent evolution and local adaptation (Fig. 3). Population differentiation based on neutral microsatellite loci (*G*_ST_) was 0.21 ± 0.02 (standard error), with pairwise values ranging from 0.002 to 0.42. Overall population differentiation in growth rate (*Q*_ST_) was 0.25 ± 0.02 at 12°C, 0.12 ± 0.01 at 18°C, and 0.41 ± 0.02 at 22°C. The pairwise *Q*_ST_ values ranged from 0.00 to 0.68 at 12°C, 0.00 to 0.60 at 18°C, and from 0.00 to 0.78 at 22°C. The overall *Q*_ST_ was significantly lower than the overall *G*_ST_ at 18°C (df = 10, *t* = 3.163, *P* ≤ 0.01), not significantly different at 12°C (df = 12, *t* = 1.38, *P* = 0.19) and significantly higher at 22°C (df = 13, *t* = 6.89, *P* ≤ 0.001).

Additive genetic variation accounted for 64–80% of the variation in growth rate at the three temperatures. Mean heritability was 0.58 at 12°C and 18°C and 0.76 at 22°C (Table). Genotype-by-temperature interactions (G × T) accounted for a portion of the phenotypic variation in all populations between 12°C and 18°C and between 18°C and 22°C (Table 4). Between the two benign temperatures, 12°C and 18°C, additive genetic variation accounted for 46% of the variation or 2.3 times more than the G × T interaction. When the stressful temperature of 22°C was compared with 18°C, G × T interaction accounted for 44% of the total phenotypic variation or 1.6 times the variation explained by the additive genetic effects.

**Table 4:** Additive genetic variance (*V*_G_), genetic variation in plasticity induced by temperature (*V*_G×T_) and environmental variance (*V*_E_) for growth under 12°C and 18°C as well as 18°C and 22°C for nine populations of *Rhynchosporium commune*. Standard errors for variance components come from jackknifing over isolates. The last lines summarize means, standard deviations (SD), and coefficients of variation (CV)

12–18°C			18–22°C		
Country						
*V*_G_ ± SE	*V*_G×T_ ± SE	*V*_E_ ± SE	*V*_G_ ± SE	*V*_G×T_ ± SE	*V*_E_ ± SE
CH	0.0649 ± 0.0020	0.0244 ± 0.0009	0.0134 ± 0.0003	0.0364 ± 0.0018	0.0367 ± 0.0012	0.0135 ± 0.0004
US	0.0231 ± 0.0007	0.0015 ± 0.0002	0.0142 ± 0.0002	0.0000 ± 0.0000	0.0550 ± 0.0018	0.0103 ± 0.0002
FI	0.0097 ± 0.0004	0.0042 ± 0.0004	0.0270 ± 0.0005	0.0010 ± 0.0004	0.0166 ± 0.0009	0.0293 ± 0.0005
ET	0.0557 ± 0.0023	0.0056 ± 0.0003	0.0203 ± 0.0006	0.0444 ± 0.0023	0.0316 ± 0.0027	0.0180 ± 0.0008
NO	0.0083 ± 0.0007	0.0160 ± 0.0006	0.0224 ± 0.0004	0.0018 ± 0.0005	0.0243 ± 0.0007	0.0270 ± 0.0005
NZ	0.0525 ± 0.0012	0.0095 ± 0.0006	0.0158 ± 0.0003	0.0306 ± 0.0011	0.0223 ± 0.0008	0.0126 ± 0.0004
SP	0.0001 ± 0.0001	0.0066 ± 0.0011	0.0445 ± 0.0018	0.0079 ± 0.0021	0.0212 ± 0.0019	0.0338 ± 0.0012
IS	0.0366 ± 0.0028	0.0195 ± 0.0012	0.0160 ± 0.0004	0.0306 ± 0.0017	0.0300 ± 0.0009	0.0090 ± 0.0002
AU	0.0070 ± 0.0013	0.0263 ± 0.0038	0.0213 ± 0.0014	0.0000 ± 0.0000	0.0108 ± 0.0009	0.0145 ± 0.0005
Mean	0.0287	0.0126	0.0217	0.0170	0.0276	0.0187
SD	0.0244	0.0092	0.0096	0.0182	0.0129	0.0090
CV	0.8518	0.7294	0.4452	1.0716	0.4686	0.4848

**Figure 4:**
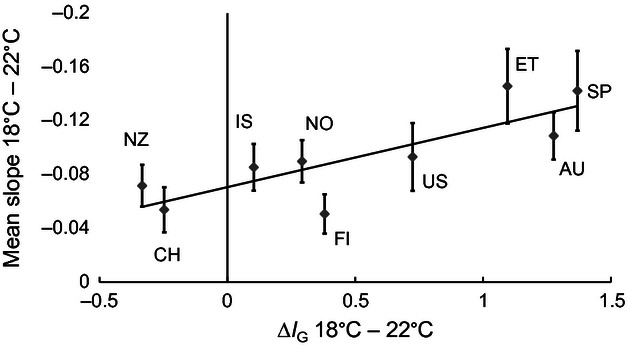
Change in trait mean-standardized additive genetic variance (delta *I*_G_) compared with level of plasticity (slope) between 18°C and 22°C. The populations that had the biggest reduction in growth rate between the two temperatures had the highest increase in *I*_G_. Error bars represent ± 1 SE.

There was a statistically significant negative correlation between trait mean-standardized levels of additive genetic variation (*I*_G_) and mean growth rate for the nine populations at all three temperatures (Table 5). This result implies that the populations that were better adapted to grow under a particular temperature had lower evolvability. The same trend was found between the two alternative measures of genetic variation, that is, heritability and additive genetic variation, and mean growth rate for the nine populations at all three temperatures. In addition, we found a significant positive correlation between the reduction in growth rate between 18°C and 22°C (mean slope) and the increase in *I*_G_ between the two temperatures (*n* = 9, *r* = 0.82, *P* ≤ 0.007) (Fig. 4), indicating that as the amount of stress populations perceived at 22°C increased, the evolvability also increased. Levels of mean-standardized additive genetic variation were higher at 22°C than 18°C in seven of the nine populations (Fig. 4). The populations from Spain, Ethiopia, and Australia showed the most pronounced decrease in growth rate between 18°C and 22°C, and they also had the highest increase in *I*_G_.

**Table 5:** Correlations between population-mean growth rate (GR) under three experimental temperatures and three measures of genetic variation: trait mean-standardized additive genetic variance (*I*_G_), additive genetic variance (*V*_G_), and heritability (*h*^2^). Sample size is nine populations

Dependent variable	GR 12°C	GR 18°C	GR 22°C
*I*_G_	−0.66*	−0.69*	−0.77*
*V*_G_	−0.46	−0.57	−0.62
*h*^2^	−0.32	−0.67*	−0.85*

* *P* ≤ 0.05.

The relationship between genetically determined plasticity and environmental canalization was explored in relation to population-mean growth rate. To determine whether G × T interactions were associated more with plasticity or environmental canalization on a population level, we looked at the association between plasticity (T) and G × T between 18°C and 22°C. Mean slopes between populations differed by up to threefold, indicating varying degrees of plasticity and canalization in different populations. However, we did not find any correlation between either the absolute G × T values (*n* = 9, *r* = −0.002, *P* = 0.99) or their fraction of total variation (*n* = 9, *r* = −0.071, *P* = 0.86) and the slope between mean population growth rate at 18°C and 22°C.

## Discussion

The ability to adapt to different thermal regimes is of great importance because temperature affects numerous traits during the lifespan of an individual, especially in ectothermic species for which there is a direct link between environmental temperature and fitness components (Angilletta 2009). Indeed, the recent literature clearly illustrates the effects of climatic conditions and especially temperature heterogeneity on fitness components of pathogens and host-pathogen coevolution (Laine 2008; Zhan and McDonald 2011; Mboup et al. 2012). Hemibiotrophic fungal pathogens of annual crops such as *R. commune* provide excellent model systems for the study of thermal adaptation because they are found in agro-ecosystems that are globally distributed across a wide range of climates. Most of these species have to overwinter or oversummer in a dormant or saprophytic state, often during times of the year exhibiting thermal extremes, yet must be able to grow, infect, and reproduce quickly during the sometimes short growing season of the host plant.

In this study, we found several lines of evidence supporting climate adaptation in *Rhynchosporium commune*. The lower two experimental temperatures of 12°C and 18°C led to significantly higher growth rate compared with 22°C, which therefore was considered stressful for the pathogen. Variability in thermal conditions at the collection sites was positively correlated with growth rate in the laboratory, particularly at 22°C. At this higher temperature, isolates from colder locations also had significantly higher growth rate than isolates from warmer locations. The results from the *Q*_ST_/*G*_ST_ comparison showed that under the more benign temperatures of 12°C and 18°C, population differentiation in growth did not reveal evidence for diversifying selection, but at 22°C, divergence provided compelling evidence for diversifying selection (Fig. 3). A significantly lower *Q*_ST_ than *G*_ST_ at 18°C can be interpreted as evidence for stabilizing selection. Stabilizing selection may also explain the absence of a significant correlation between temperature variables at the field sites and growth at 18°C. A likely explanation is that 18°C provides growth conditions that are close to optimal for all populations across the species. *Q*_ST_ at 12°C did not deviate significantly from *G*_ST_, indicating that the hypothesis of neutral evolution for this trait cannot be rejected. We hypothesize that the observed pattern for growth rate at the three temperatures can be explained by a combination of several factors: i) Isolates from host populations in more variable climates benefit from the ability to grow rapidly during the relatively shorter host growing season, even under high temperatures. ii) Temperature variation favors isolates with relatively high growth rate over a wide temperature range. iii) Selection for high growth rate is weaker in locations with a constant climate where the host is growing for longer periods and optimum temperatures are more frequent. These explanations also indicate a possible trade-off for high growth rate at above-optimum temperatures in locations with constant climates.

As our experiments did not allow us to test whether variable environments induce generally low growth rate, this environmental gradient is not suitable for identifying counter-gradient variation without further experiments. Information on whether populations from variable climates have relatively higher growth rate across the full range of thermal variation would be needed to draw robust conclusions about the pattern of thermal adaptation. However, we hypothesize that if highly variable environments reflect locations with shorter host growing seasons, pathogen isolates may have evolved faster growth to compensate for these environmental effects and this could be considered counter-gradient variation. The negative correlation between mean annual temperature and growth rate at 22°C (Fig. 2A) also points toward counter-gradient variation where poor growth conditions led to evolution of a rapid growth rate. Counter-gradient variation has so far rarely been reported in fungi. McLean et al. (2005) found evidence for both a shift in thermal performance measured as colony growth and counter-gradient variation in four fungal species. A recently published study of another fungal leaf pathogen, *Mycosphaerella graminicola,* found thermal adaptation consistent with a shift in thermal performance for growth corresponding to the local temperature regime (Zhan and McDonald 2011).

Mean estimates of additive genetic variation and heritability for growth rate were high in all environments (Table). This finding is in line with results from other fungal plant pathogens (Willi et al. 2010, 2011; Zhan and McDonald 2011). Both mean heritability and evolvability were highest under thermal stress at 22°C, compared with the other, more benign temperatures. Genetic variation accounted for more of the total phenotypic variation than residual variation (*V*_E_) in all nine populations at 22°C. These results agree with studies on fungal pathogens which found increased genetic variation under stressful compared to benign environmental conditions (Willi et al. 2010, 2011). We hypothesize that the increase in genetic variation in response to heat stress in seven of the nine populations is due to de-canalization following a history of selection for canalization in more favorable environments (Hoffmann and Merilä 1999). Thermal stress related to de-canalization has been associated with reduced functioning of heat shock proteins in *Drosophila* species (Rutherford and Lindquist 1998) which triggered the expression of novel phenotypes. Further experiments will be needed to differentiate among other hypotheses that can explain an increase in genetic variation under stress (Hoffmann and Merilä 1999), but we believe that the novel-stress hypothesis is an unlikely explanation in this case because the pathogen is likely to be exposed to 22°C during the host growing season in all populations included in this study.

Interestingly, all three estimates of genetic variation were lower in populations with higher growth rate at all temperatures (Table 5). We offer two potential explanations for these findings. First, fluctuating selection in populations in more variable climates with higher growth rates could have lowered genetic variation. This has been found in evolution experiments in both *Callosobruchus maculatus* (Hallsson and Björklund 2012) and *Drosophila melanogaster* (Pelabon et al. 2010). The second explanation is that if variable climates reflect locations with short growing seasons, then populations in those locations might have been under strong directional selection for more rapid growth. Past directional selection has been linked with the fixation of favored alleles and thus lower genetic variation (Blows and Hoffmann 1993).

Our finding of a negative correlation between genetic variation and growth rate is in contrast with some other studies. Kellermann et al. (2009) found a positive correlation between trait mean and genetic variation for cold and desiccation resistance in a species-level study including multiple *Drosophila* species. Furthermore, the authors found that species with low trait means for both resistance traits had a more restricted distribution compared with species with higher trait means, which led them to conclude that low genetic variation is responsible for species distribution limits. Other studies have reported similar findings (Bradshaw 1991; Hoffmann et al. 2003), while for example Sgrò and Blows (2003) identified two peaks in additive genetic variance associated with low and high trait means across the range of values for mean developmental time in 10 populations of a *Drosophila* species. The relationship between trait means and genetic variances seems to be complex and impacted by several factors.

Variation among isolates in growth rate across temperatures provided evidence for considerable genetically determined plasticity and environmental canalization. We found that genotype-by-temperature interactions (G × T) explained a portion of the variation in growth rate, both between 12°C and 18°C and between 18°C and 22°C (Table 4). Such genetic variation for phenotypic plasticity and environmental canalization is another contributor to adaptability of growth rate across environments (reviewed in Ghalambor et al. 2007). We further investigated the relative contribution of G × T compared with both additive genetic variation and plasticity/canalization. G × T accounted for more variation than (temperature-independent) genetic effects between 18°C and 22°C, but it accounted for much less variation than genetic effects between 12°C and 18°C. Finally, we found no association between G × T and plasticity (T) in response to above-optimal temperatures at the population level. We interpret this finding as evidence that past selection for plasticity has not exhausted genetic variation for plasticity and that populations with high levels of plasticity are not constrained in their future response to selection for increased plasticity.

In this study we have argued that to predict the future distribution of a species under climate warming, we need to simultaneously assess past thermal adaptation, genetic variation needed for future adaptation, and how they are linked. For our study organism *Rhynchosporium commune*, we found evidence for local adaptation to stressful, high temperature as well as mean annual temperature variation in addition to a high adaptive potential for growth rate at all experimental temperatures in most populations. Genetic variation was lower under benign than stressful temperatures and lower in populations with higher growth rate, presumably due to some depletion of genetic variation by selection. This, along with evidence for extensive genetic variation in plasticity in response to thermal extremes, leads us to conclude that the species distribution will not be limited by a lack of genetic variation under climate warming. Future studies on the potential for thermal adaption in *R. commune* and other plant pathogens should include a larger fraction of the thermal performance curve, performance under variable conditions, and additional elements of species fitness such as virulence and reproductive output.

## Data archiving statement

Data for this study are available at: Dryad - doi:10.5061/dryad.0 h887
